# Preoperative systemic inflammation response index may predict postoperative delayed extubation for elderly patients with aSAH: a retrospective cohort study

**DOI:** 10.1186/s12883-025-04457-1

**Published:** 2025-11-03

**Authors:** Mingchao Fan, Xuehua Xiong, Shifang Li, Lei Cheng, Yugong Feng, Qiaoling Wang, Guifeng Yang

**Affiliations:** 1 Department of Neurosurgery, The Affiliated Hospital of Qingdao University, Qingdao, China; 2 Department of Neurosurgery, Chongqing Emergency Medical Center, Chongqing University Central Hospital, Chongqing, China; 3 Department of Neurosurgical Intensive Care Unit, The Affiliated Hospital of Qingdao University, Qingdao, China; 4 The Community Medical Service Center of Zhenjiang Street, North City District, Qingdao, China; 5 Department of Radiology, the Third People’s Hospital of Qingdao, Qingdao, China

**Keywords:** Systemic inflammation response index, Delayed extubation, Elderly, Lactic dehydrogenase, Aneurysmal subarachnoid hemorrhage

## Abstract

**Background:**

Aneurysmal subarachnoid hemorrhage (aSAH) is one common neurosurgical emergency and severe disease, with high morbidity and mortality. Delayed extubation (DE) is a confirmed risk factor for prolonged intensive care unit (ICU) stay and poor outcomes in patients undergoing neurosurgical operation. The systemic inflammation response index (SIRI) has emerged as a novel, valuable prognostic marker for various neoplastic and critical conditions.

**Objective:**

This study aimed to investigate the association between pre-operative SIRI and post-operative DE in elderly patients underwent neurosurgical clipping or endovascular coiling for aSAH.

**Methods:**

We conducted a retrospective analysis of elderly aSAH patients underwent neurosurgical clipping or endovascular coiling between Jan 2016 and Dec 2022. Patient epidemiologics, clinical variables, and potential influencing factors were assessed. Multivariate logistic regression and receiver operating characteristics (ROC) curve analyses were employed to determine SIRI’s predictive value for DE.

**Results:**

Among the 413 enrolled patients, 113 patients experienced DE, while 300 patients did not. Multivariate logistic regression analysis identified GCS (OR, 0.631; 95% CI, 0.454–0.879; *P* = 0.006); lactic dehydrogenase (OR, 1.008; 95% CI, 1.001–1.016; *P* = 0.042), and SIRI (OR, 1.171; 95% CI, 1.050–1.328; *P* = 0.006) as independent predictors of DE. ROC curve analysis demonstrated that SIRI effectively predicted postoperative DE (AUC = 0.772, 95% CI, 0.696–0.849, *P* < 0.001). The optimal SIRI cutoff value was 8.14, yielding a sensitivity of 42.0% and specificity of 95.0%.

**Conclusion:**

Pre-operative SIRI is an independent risk factor for post-operative DE in elderly patients undergoing neurosurgical operation for aSAH.

## Introduction

Aneurysmal subarachnoid hemorrhage (aSAH) is a devastating subtype of hemorrhagic stroke, and is associated with high morbidity and mortality, at least 25% of patients die, and more than 50% survivors suffer from residual neurological deficits [1, 2]. Although individuals aged 40–60 years constitute the primary demographic for aSAH, the aging of society has led to a growing number of elderly patients with aSAH, which has attracted widespread attention [3, 4]. Advanced age is considered as an independent risk factor for un-favorable outcomes in various critical diseases, including aSAH [5, 6]. Frailty and impaired physical function in elderly patients are significantly associated with prolonged hospital stays and severe complications, often resulting in poor prognosis and increased mortality [7, 8]. However, aSAH-related parameters derived from studies primarily focusing on middle-aged populations have notable limitations when applied to elderly patients. Research on aSAH in this elderly population remains limited, necessitating further investigation of additional relevant parameters.

Artificial airway ventilation is a key therapeutic measure for improving inadequate ventilation in critically ill patients, encompassing invasive mechanical ventilation and spontaneous breathing with an artificial airway. The establishment of an artificial airway is the foundation for ensuring ventilation during general anesthesia. Owing to disease complexity and certain postoperative complications, aSAH often results in prolonged artificial airway ventilation. While most aSAH patients can recover spontaneous breathing and have their tracheal tube removed promptly after surgery, early extubation remains challenging in some cases. Multiple factors contribute to delayed extubation (DE), including reduced level of consciousness, respiratory muscle weakness, impaired airway self-cleaning ability, and increased respiratory secretions [9]. Previous studies have indicated that prolonged artificial airway ventilation is significantly associated with poor prognosis in patients undergoing general anesthesia [10, 11]. DE not only extends the intensive care unit (ICU) stay, but also elevates the risk of hospital-acquired pneumonia (HAP) and increases medical costs [12, 13].

The systemic inflammation response index (SIRI), calculated using Peripheral blood routine test parameters, is a novel comprehensive inflammatory index that better reflects the body’s inflammatory state [14]. Numerous studies have shown that SIRI holds significant value for prognostic assessment in various malignancies and other critical illnesses [15–17]. To the best of our knowledge, SIRI has not been reported as a predictive factor for DE, particularly in elderly patients undergoing neurosurgical operation for aSAH. We hypothesized that preoperative SIRI could predict DE in patients undergoing neurosurgical operation for aSAH. In this study, we sought to analyze the association between preoperative SIRI and postoperative DE in elderly patients undergoing neurosurgical operation for aSAH.

## Methods

### Patient population

This retrospective cohort study enrolled elderly patients admitted to the Affiliated Hospital of Qingdao University between Jan 2016 and Dec 2022, who were diagnosed with aSAH underwent neurosurgical clipping or endovascular coiling.

The Inclusion criteria were as follows: aged ≥ 60 year-old; diagnosed with aSAH; underwent neurosurgical clipping or endovascular coiling therapy; time from symptom onset to surgery < 72 h; no preoperatively indwelling artificial airway (artificial airway establishment within 2 h before surgery was classified as part of the surgical procedure); no immunosuppressant or hormone therapy within 3 months prior to admission; complete medical record data.

The exclusion criteria were as follows: presence of hematologic diseases other than iron deficiency anemia; history of malignant tumors, autoimmunediseases or infectious diseases; history of stroke, traumatic brain injury, or craniotomy; presence of severe respiratory disease (e.g., chronic obstructive pulmonary disease); loss to follow-up.

### Data collection

Basic population data and admission clinical information were collected and recorded from the hospital information system of The Affiliated Hospital of Qingdao University, including age, gender, past medical history, smoking and drinking history, body mass index (BMI), blood pressure, body temperature, heart rates, respiratory rates, Glasgow Coma Scale (GCS) score [18], Hunt-Hess grade [19], aSAH imaging information (modified Fisher grade; number, size, location of aneurysms; ventricle hematoma; and hydrocephalus), complete blood cell count, anesthesia duration, surgical duration, blood biochemical tests, and coagulation function tests. Additionally, information on surgical approach, type and duration of postoperative artificial airway ventilation, total hospital stay and ICU stay was recorded.

All patients received craniocerebral computer tomography (CT) and computed tomography angiography (CTA) as soon as possible after admission to confirm the diagnosis. Digital subtraction angiography (DSA) was performed if necessary. Aneurysm size was defined by the maximum diameter; for patients with multiple aneurysms, the largest one was used for analysis. Aneurysms were categorized by maximum diameter as follows: small (≤ 5 mm), medium (6–15 mm), large (16–24 mm), and giant (≥ 25 mm). They were also classified by origin location of origin: arteriae cerebri anterior, middle cerebral artery, internal carotid artery, anterior communicating artery, posterior communicating artery and posterior circulation arteries. Aneurysm size and location were confirmed by at least two neurosurgeons and one radiologist. The diagnosis of Ventricle hematoma and hydrocephalus was made by radiologists based on craniocerebral CT results.

The surgical approach was determined by neurosurgeons in discussion with the patient’s guardians, based on the patient’s condition and imaging characteristics. All patients received general anesthesia with mechanical ventilation via oral tracheal intubation; nasal endotracheal intubation was considered if oral intubation was contraindicated. Post-anesthesia recovery was condition in either the neurosurgical intensive care unit (NICU), depending on the patient’s condition. Prolonged artificial airway ventilation was determined by the neurocritical physician based on the comprehensive assessment of the patient’s consciousness level and respiratory status. Daily extubation assessments were performed; if a patient was expected to require intubation for more than 2 weeks, temporary tracheotomy was performed with the guardian consent. All artificial airway ventilation included necessary heating and humidification of inhaled gas. Oxygen concentration was adjusted according to clinical needs, with avoidance of unnecessarily high concentrations. The decision to initiate mechanical ventilation and adjustments to ventilator parameters were made by the neurocritical care physician based on patient needs.

SIRI was calculated using the formula: SIRI = monocyte count × neutrophil count/lymphocyte count. DE was defined as tracheal intubation lasting >48 h after surgical operations, or re-intubation within 48 h after extubation. The modified Rankin Scale (mRS) [20] was used to evaluate the prognosis at 30 days after surgery. A score of 0–2 indicated a good prognosis, while 3–6 indicated a poor prognosis.

### Statistical analysis

Statistical analysis were performed using IBM SPSS Statistics 27.0 (SPSS Inc., Chicago, Illinois, USA). Continuous variables were presented as means ± standard deviation (SD) for normally distributed data, or median and interquartile range (IQR, 25th to 75th percentile) for non-normally distributed data. Categorical variables were expressed as frequency (percentage). For group comparisons: the Chi-square test was used for categorical data; the Student’s t-test was used for normal distribution continuous variables; and the Kruskal-Wallis H test was used for non-normally distribution continuous variables.

Univariate and multivariate logistic regression analysis were conducted to identify independent risk factors for DE. The likelihood ratio test was used to determine whether interaction terms in the model affected model fitting. Receiver operating characteristic (ROC) curve analysis was performed to calculate the area under the curve (AUC), optimal cutoff value, sensitivity and specificity of SIRI. To align the directions of ROC curves for SIRI, GCS and lactate dehydrogenase. Use the GCS prediction probability values to replace GCS when creating the ROC curve. To verify the consistency between observed and predicted data, the Hosmer-Lemeshow goodness-of-fit test was applied to generate a calibration plot for the ROC analysis. Statistical significance was defined as *P* < 0.05.

Since SIRI includes monocyte, neutrophil and lymphocyte counts, these individual cell counts were excluded from the multivariate logistic regression models to avoid multicollinearity bias.

## Results

### Baseline characteristics

**Fig. 1:**
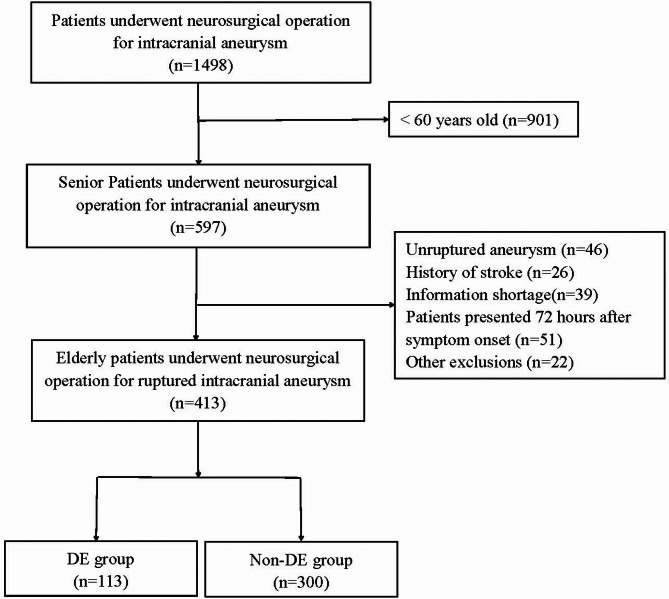
The study flow chart. (mRS: the Modified Rankin Scale)

A total of 413 patients were enrolled in this study (Fig. 1). The median age was 68 years (IQR, 65–73 years), and 316 patients (76.51%) were female. DE occurred in 113 patients (27.36%), who were assigned to the DE group; the remaining 300 patients (72.64%) constituted the non-DE group. Among patients in the DE group, 27 patients (23.89%) required reintubation within 72 h, and 51 patients (45.13%) underwent percutaneous tracheotomy. HAP was observed in 65 patients (15.74%) overall, with a significantly higher incidence in the DE group than in the non-DE group (44/113 vs. 21/300, χ^2^ = 63.136, *P* < 0.001). The 30-day postoperative mortality rate was 4.84% (*n* = 20). Aneurysm treatment consisted of neurosurgical clipping (38.01%, *n* = 157) and endovascular coiling (61.99%, *n* = 256); no significant association was found between the type of procedure and the occurrence of DE (χ^2^ = 0.451, *P* = 0.501). Detailed demographic data are presented in Table 1.

**Table 1:** The demographic and baseline characteristics

Variable	Total (*n* = 413)	Prolonged artificial airway ventilation		*P*-value
		YES (*n* = 113)	NO (*n* = 300)	
Gender (female)	316 (76.51%)	79 (69.91%)	237 (79.00%)	0.052
Age (years)	68 (65–73)	69 (66–74)	68 (64–72)	**0.021**
BMI	24.20 (22.00–26.60.00.60)	23.80 (21.53–26.38)	24.20 (22.20–26.60)	0.436
Heart rates (times/min)	76 (66–82)	77 (63–82)	76 (66–82)	0.763
Respiratory rates (times/min)	18 (16–20)	18 (16–20)	18 (16–20)	0.517
Body temperature (℃)	36.70 (36.50–37.00)	36.80 (36.40–37.00)	36.70 (36.50–37.00)	0.791
Smoking (%)	62 (15.01)	23 (20.35)	39 (13.00)	0.064
Drinking (%)	50 (12.11)	17 (15.04)	33 (11.00)	0.261
Hypertension (%)	242 (58.60)	68 (60.18)	174 (58.00)	0.775
Diabetes (%)	42 (10.17)	13 (11.50)	29 (9.67)	0.582
Cardiac insufficiency (%)	51 (12.35)	17 (15.04)	34 (11.33)	0.307
Systolic Pressure (mmHg)	147 ± 23	147 ± 24	147 ± 23	0.830
Diastolic Pressure (mmHg)	82 ± 13	81 ± 13	82 ± 13	0.125
Length of ICU stay (days)	1.0 (0.0–6.0)	11.0 (7.0–19.5.0.5)	1.0 (0.0–2.0)	**< 0.001**
Length of stay (days)	13.0 (10.0–18.0)	21.0 (21.0–32.5.0.5)	13.0 (10.0–16.0)	**< 0.001**
30-day prognosis				**< 0.001**
mRS 0–2	250 (60.53)	7 (6.19)	243 (81.00)	
mRS 3–6	163 (39.47)	106 (93.81)	57 (19.00)	
GCS	14 (11–15)	9 (7–13)	15 (14–15)	**< 0.001**
Fisher Grade	2 (2–3)	3 (2–4)	2 (2–2)	**< 0.001**
Hunt-Hess Grade	2 (1–3)	4 (3–4)	2 (1–3)	**< 0.001**
Hunt-Hess Grade distribution (%)				**< 0.001**
Grade Ⅰ	112 (27.12)	12 (10.62)	100 (33.33)	
Grade Ⅱ	134 (32.45)	11 (9.73)	123 (41.00)	
Grade Ⅲ	76 (15.40)	19 (16.81)	57 (19.00)	
Grade Ⅳ	77 (18.64)	57 (50.44)	20 (6.67)	
Grade Ⅴ	14 (3.39)	14 (12.39)	0 (0.00)	
Location of aneurysm (%)				0.277
ACA	27 (6.54)	5 (4.42)	22 (7.33)	
ICA	121 (29.30)	35 (30.97)	86 (28.67)	
MCA	88 (21.31)	24 (21.24)	64 (21.33)	
ACoA	82 (19.85)	17 (15.04)	65 (21.67)	
PCoA	68 (16.46)	23 (20.35)	45 (15.00)	
Posterior circulation arteries	27 (6.54)	9 (7.96)	18 (16.00)	
Aneurysm size (mm)	5.30 (4.00–8.00)	6.00 (4.00–9.00)	5.03 (4.00–7.11.00.11)	0.058
Aneurysm size distribution (%)				0.286
Small (≤ 5 mm)	161 (38.98)	47 (41.59)	114 (38.00)	
Medium (6–15 mm)	241 (58.35)	63 (55.75)	178 (59.33)	
Large (16–24 mm)	7 (1.69)	3 (2.65)	4 (1.33)	
Giant (≥ 25 mm)	4 (0.96)	0 (0.00)	4 (1.33)	
Aneurysm number (%)				0.713
= 1	371 (89.83)	102 (90.27)	269 (89.67)	
≥ 2	42 (10.17)	11 (9.73)	31 (10.33)	
Operation method (%)				0.501
Clipping	256 (61.99)	73 (64.60)	183 (61.00)	
Coiling	157 (38.01)	40 (35.40)	117 (39.00)	
Anesthesia duration	4.78 (3.87–6.15)	5.11 (4.32–6.98)	4.76 (4.08–6.67)	0.190
Surgical time	3.42 (2.51–4.01)	3.67 (2.61–4.09)	3.2 (2.33–3.97)	0.274
Ventricle hematocele (%)	228 (55.21)	89 (78.76)	139 (46.33)	**< 0.001**
Hydrocephalus (%)	244 (59.08)	90 (79.65)	154 (51.33)	**< 0.001**
WBCs (×10^9^/L)	10.30 (7.79–12.86)	12.51 (10.39–16.31)	9.33 (7.44–12.11)	**< 0.001**
RBCs (×10^12^/L)	4.30 ± 0.53	4.23 ± 0.67	4.33 ± 0.46	0.074
Hemoglobin (g/L)	130.77 ± 16.05	129.25 ± 19.66	131.34 ± 14.45	0.239
Monocyte (×10^9^/L)	0.53 (0.37–0.74)	0.63 (0.41–0.89)	0.50 (0.36–0.67)	**< 0.001**
Neutrophil (×10^9^/L)	8.30 (5.87–11.16)	10.45 (8.23–14.41)	7.46 (5.28–9.97)	**< 0.001**
Lymphocyte (×10^9^/L)	1.18 (0.85–1.65)	1.03 (0.79–1.56)	1.22 (0.89–1.67)	**0.016**
Platelet (×10^9^/L)	210 (174–244)	217 (173–254)	207 (174–242)	0.225
Serum albumin (g/L)	38.99 ± 4.94	38.76 ± 5.66	39.08 ± 4.65	0.601
Serum Glucose (mmol/L)	7.07 (5.82–9.58)	8.02 (6.80–10.47.80.47)	6.70 (5.57–7.94)	0.821
Lactic dehydrogenase (U/L)	181.50 (154.70–213.75.70.75)	222.50 (168.25–281.00)	173.00 (151.00–198.25.00.25)	**< 0.001**
Urea nitrogen (mmol/L)	4.93 (4.00–6.20.00.20)	5.65 (4.22–6.96)	4.80 (3.82–5.92)	**< 0.001**
Creatinine (umol/L)	60.00 (46.20–73.00)	57.85 (46.48–77.95)	60.40 (46.00–71.00)	0.259
Serum potassium (mmol/L)	3.79 ± 0.44	3.72 ± 0.45	3.82 ± 0.43	**0.041**
Serum natrium (mmol/L)	139.58 ± 4.40	140.03 ± 5.49	139.41 ± 3.92	0.211
Fibrinogen (g/L)	3.10 (2.65–3.61)	3.20 (2.63–3.67)	3.08 (2.66–3.56)	0.417
SIRI	3.44 (1.82–6.12)	5.72 (3.22–10.70)	2.91 (1.60–5.11)	**< 0.001**

*BMI* Body mass index, *GCS* Glasgow coma scale, *SIRI* Systemic inflammation response index, *WBCs* White blood cell, *RBCs* Red blood cells, *mRS* the modified Rankin Scale, *ACA* Arteriae cerebri anterior, *ICA* Internal carotid artery, *MCA* Middle cerebral artery, *ACoA *Arteriae communicating anterior, *PCoA* Posterior communicating artery

The significance of bold data: *P* < 0.05

### Relationship between DE and prognosis

**Fig. 2:**
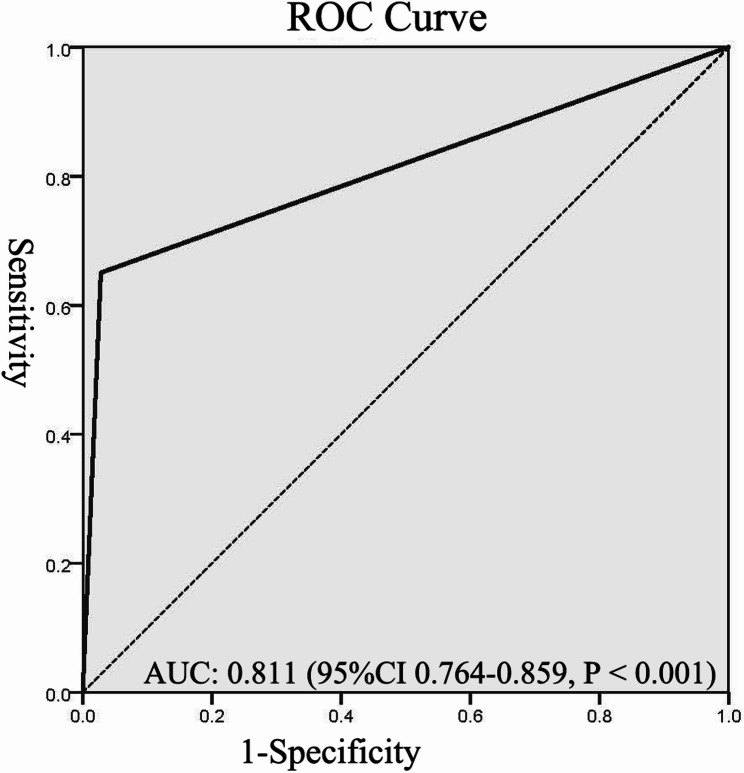
The receiver-operating characteristic curve for delayed extubation. (AUC: the area under the curve)

Among the 113 patients with DE, 106 patients (93.81%) had poor 30-day postoperative outcomes, whereas only 7 patients (6.19%) had favorable outcomes. This was in sharp contrast to the non-DE group, in which favorable outcomes were significantly more common than poor outcomes (243 cases vs. 57 cases; χ²=192.259, *P* < 0.001). Both total hospitalization duration (Z = −6.349, *P* < 0.001) and ICU length of stay (Z = −14.937, *P* < 0.001) were significantly prolonged in the DE group relative to non-DE counterparts. Univariate logistic regression analysis showed that DE was a significant risk factor for poor 30-day prognosis in elderly aSAH patients (OR = 64.556; 95%CI: 28.505–146.202.505.202; *P* < 0.001). The ROC curve analysis demonstrated DE’s predictive capacity for poor neurosurgical outcomes in this population (AUC = 0.811; 95%CI: 0.764–0.859; *P* < 0.001) (Fig. 2). At the optimal cutoff value of 0.5, sensitivity reached 65.0% with specificity of 97.2%.

### Univariate and multivariate logistic regression analyses

Univariate logistic regression analysis identified several factors significantly associated with postoperative DE in elderly aSAH patients underwent neurosurgical operation: age (OR, 1.046; 95% CI, 1.005–1.089; *P* = 0.026); GCS (OR, 0.632; 95% CI, 0.578–0.691; *P* < 0.001); Fisher grade (OR, 3.998; 95% CI, 2.895–5.520; *P* < 0.001); Hunt-Hess grade (OR, 3.678; 95% CI, 2.822–4.793; *P* < 0.001); ventricle hematocele (OR, 4.471; 95% CI, 2.688–7.438; *P* < 0.001); hydrocephalus (OR, 3.877; 95% CI, 2.319–6.480; *P* < 0.001); serum glucose (OR, 1.325; 95% CI, 1.204–1.458; *P* < 0.001); lactic dehydrogenase (OR, 1.016; 95% CI, 1.010–1.022; *P* < 0.001); serum potassium (OR, 0.588; 95% CI, 0.352–0.980; *P* = 0.042); SIRI (OR, 1.233; 95% CI, 1.159–1.313; *P* < 0.001).

Factors with significant associations in the univariate analysis were included in the multivariate logistic regression analysis, which identified three independent risk factors: GCS (OR, 0.631; 95% CI, 0.454–0.879; *P* = 0.006); lactic dehydrogenase (OR, 1.008; 95% CI, 1.001–1.016; *P* = 0.042); and SIRI (OR, 1.171; 95% CI, 1.050–1.328; *P* = 0.006). Complete regression results are presented in Table 2.

**Table 2:** Univariate and multivariate regression analysis of factors related to delayed extubation

Predictors	Univariate analysis		Multivariate analysis	
	OR (95% CI)	*P*-value	OR (95% CI)	*P*-value
Age (years)	1.046 (1.005–1.089)	**0.026**	1.009 (0.928–1.097)	0.827
GCS	0.632 (0.578–0.691)	**< 0.001**	0.631 (0.454–0.879)	**0.006**
Fisher Grade	3.998 (2.895–5.520)	**< 0.001**	1.731 (0.880–3.405)	0.112
Hunt-Hess Grade	3.678 (2.822–4.793)	**< 0.001**	0.670 (0.286–1.569)	0.357
Ventricle hematocele	4.471 (2.688–7.438)	**< 0.001**	1.142 (0.398–3.279)	0.805
Hydrocephalus	3.877 (2.319–6.480)	**< 0.001**	1.499 (0.518–4.342)	0.455
Serum Glucose (mmol/L)	1.325 (1.204–1.458)	**< 0.001**	1.034 (0.866–1.234)	0.715
Lactic dehydrogenase	1.016 (1.010–1.022)	**< 0.001**	1.008 (1.001–1.016)	**0.042**
Serum potassium	0.588 (0.352–0.980)	**0.042**	0.836 (0.285–2.454)	0.744
SIRI	1.233 (1.159–1.313)	**< 0.001**	1.171 (1.050–1.328)	**0.006**

*GCS* Glasgow coma scale, *SIRI* Systemic inflammation response index

The significance of bold data: *P* < 0.05

### ROC curve analysis

**Fig. 3:**
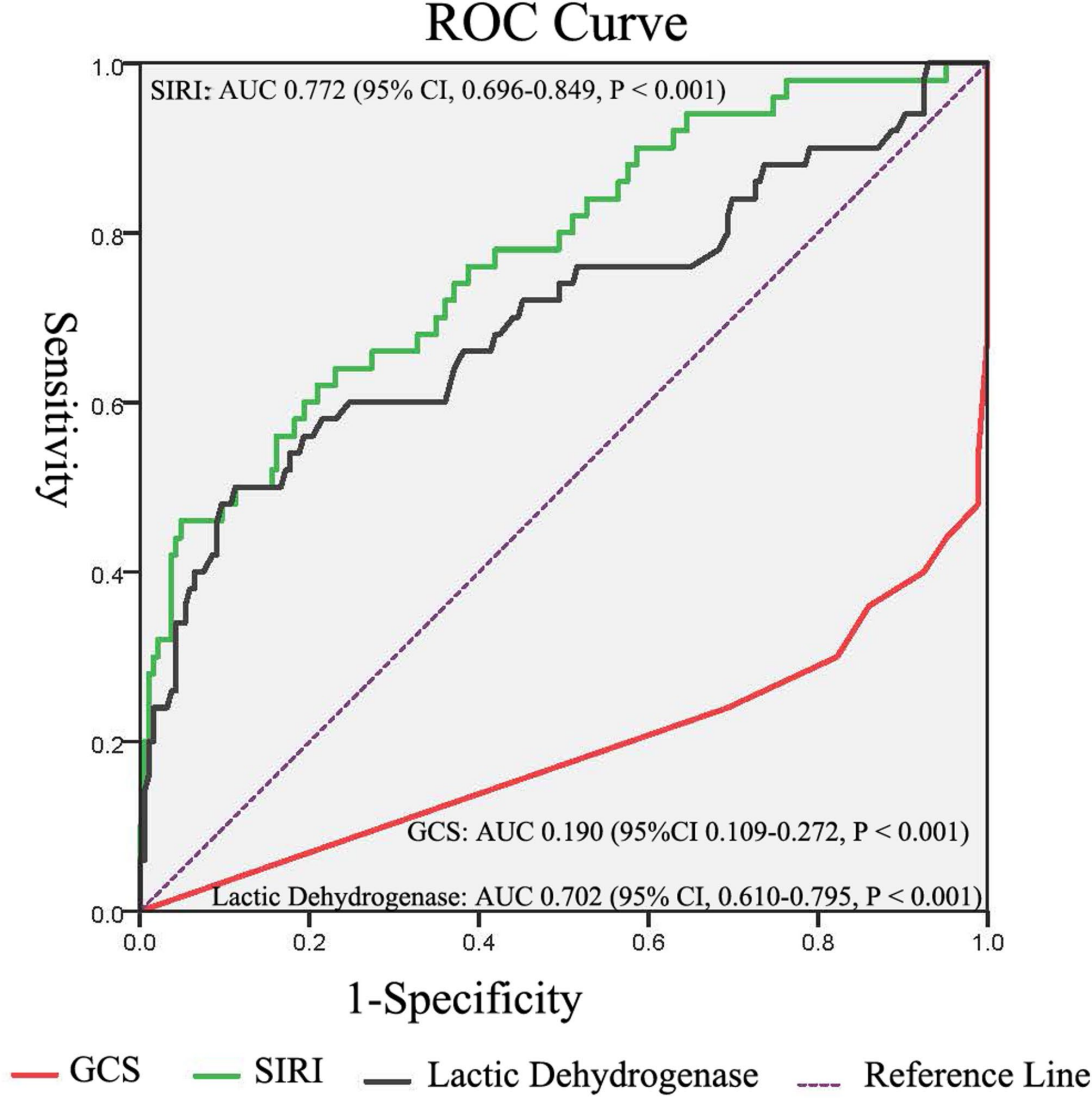
The receiver-operating characteristic curves. ROCs for GCS, SIRI and lactic dehydrogenase. (GCS Glasgow Coma Scale, SIRI systemic inflammation response index, ROC receiver operating characteristic curve)

**Fig. 4:**
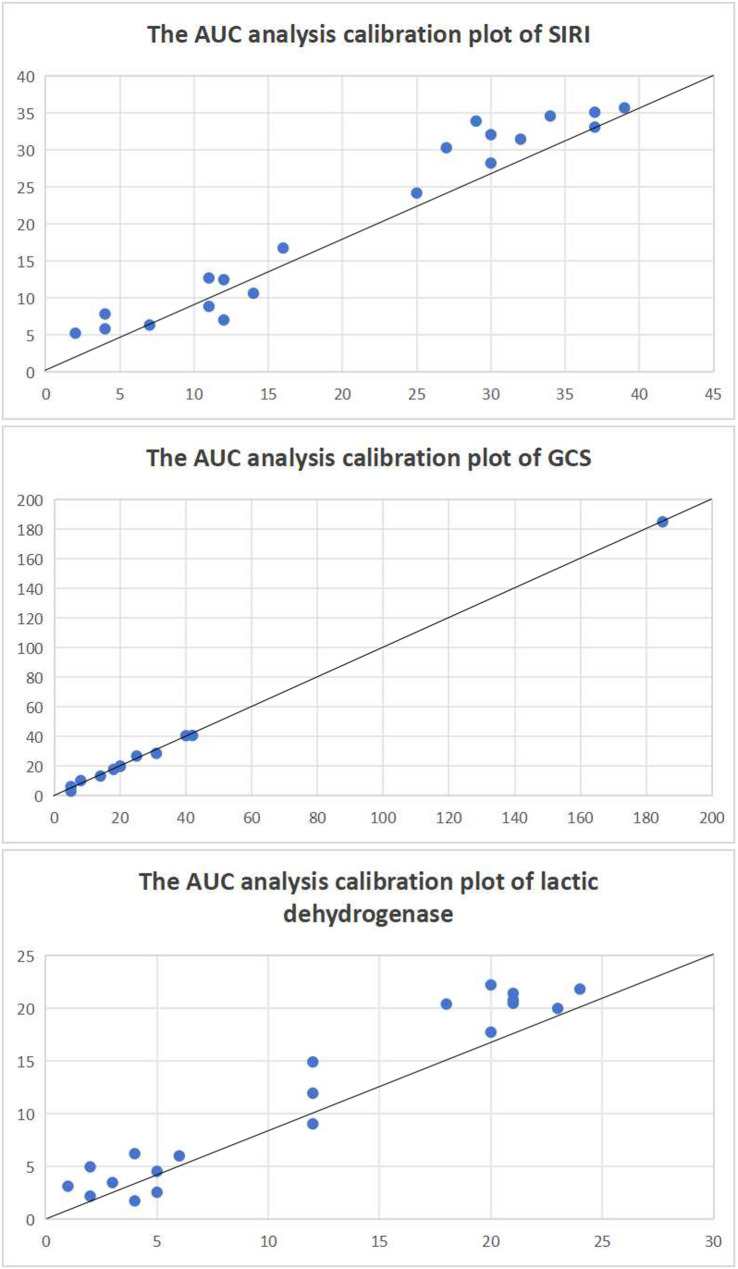
The AUC analysis calibration plots for GCS, SIRI and lactic dehydrogenase

ROC curve analysis assessed the predictive performance of GCS, lactic dehydrogenase and SIRI for postoperative DE in elderly aSAH patients underwent neurosurgical operation. The corresponding AUC of GCS was 0.190 (95% CI, 0.109–0.272, *P* < 0.001), with the cutoff value was 11.5 (sensitivity 93.0%, specificity 60.0%). The Hosmer-Lemeshow goodness-of-fit test for GCS showed χ^2^ = 2.305 (*P* = 0.680). The corresponding AUC of lactic dehydrogenase was 0.702 (95% CI, 0.610–0.795, *P* < 0.001), with the cutoff value was 229.5 (sensitivity 50.0%, specificity 89.0%). The Hosmer-Lemeshow goodness-of-fit test for lactate dehydrogenase showed χ^2^ = 12.353 (*P* = 0.136). The corresponding AUC of SIRI was 0.772 (95% CI, 0.696–0.849, *P* < 0.001), with the cutoff value was 8.14 (sensitivity 42.0%, specificity 95.0%). The Hosmer-Lemeshow goodness-of-fit test for SIRI showed χ^2^ = 12.098 (*P* = 0.147). Complete regression results are presented in Fig. 3; Table 3. The AUC analysis calibration plots are shown in Fig. 4.

**Table 3:** Diagnostic values of factors related to delayed extubation

Variable	AUC (95% CI)	*P*-value	Cutoff Value	Sensitivity	Specificity
GCS	0.810 (0.109–0.272)	**< 0.001**	11.5	0.93	0.60
Lactic dehydrogenase	0.702 (0.610–0.795)	**< 0.001**	229.5	0.50	0.89
SIRI	0.772 (0.696–0.849)	**< 0.001**	8.14	0.42	0.95

Use the GCS prediction probability values to replace GCS when creating the ROC curve

*GCS *Glasgow coma scale,* SIRI *S*y*stemic inflammation response index

The significance of bold data: *P* < 0.05

## Discussion

In this single-center retrospective study, we analyzed the clinical and prognostic data of 413 elderly patients who underwent surgical treatment for aSAH. The first step of our work confirmed that DE is a risk factor for poor outcomes and prolonged hospital stay. We analyzed preoperative and intra-operative clinical indicators to predict the risk of postoperative DE. The result showed that preoperative GCS, lactate dehydrogenase, and SIRI were significantly associated with postoperative DE in elderly patients underwent surgical treatment for aSAH. To the best of our knowledge, this is the first report to focus on the analysis of risk factors for DE in these patients. The GCS is a conventional standard prognostic indicator of aSAH, while SIRI and lactate dehydrogenase are novel indicators that have not been reported before. Early development of intervention strategies is conducive to optimizing patient outcomes. These findings may facilitate the early prediction of postoperative DE and help avoidance of extubation failure. Early and effective predictive models for DE can assist medical staff in formulating comprehensive treatment plans and help patients’ families in coping with this challenge.

At present, the time definition of DE after surgery is still not unified standard, while the majority of clinical practitioners accepted the definition of 24–48 h [12, 21, 22]. In the present cohort study, we defined DE as the requirement for endotracheal intubation over 48 h postoperatively. The incidence of DE after general anesthesia surgery varies greatly in different diseases. Few reports of DE after craniocerebral surgery have been retrieved, and the incidence of DE after neurosurgery was 10.4%−18% [23, 24]. In our series of aSAH elderly cases who underwent neurosurgical clipping or endovascular coiling, the incidence of DE after general anesthesia was 27.36%. The relatively high incidence of DE may be associated with the characteristics of our study population; advanced age is one of the important reasons for extubation failure [25, 26].

The decrease in consciousness level is one of the important reasons of artificial airway dependence in patients with brain function injuries [25, 27]. preoperative reduced consciousness usually predicts severe aSAH, and decrease central respiratory drive, resulting in decreased airway protective reflexes and respiratory dynamic ability [28, 29]. In this study, lower preoperative GCS was an independent risk factor for postoperative DE in patients with aSAH. Patients with preoperative GCS < 12 were prone to DE after surgery. We also compared the effect of neurosurgical clamping and endovascular curling on postoperative DE, and found that there was no significant difference in the occurrence of DE between the two surgical methods (χ^2^ = 0.451, *P* = 0.501). This finding helps exclude the potential impact of the surgical procedure itself on DE development.

Similar to other acute central nervous system pathological events, aSAH also caused significant inflammatory and immune responses in both the intracranial and peripheral systems, with the response intensity being significantly correlated with the severity of the intracranial lesions [30, 31]. Aneurysm rupture leads to blood extravasations into the subarachnoid space; subsequent breakdown of plasma and blood cells releases numberous inflammatory stimuli, activates microglia, recruits neutrophils, and triggers the downstream inflammatory cascades [32–34]. Neutrophils are among the first innate immune cells to respond to brain injury and are key mediators of the inflammatory response. Their adhesion to endothelial cells has been shown to be the basis of intracranial local inflammation and the beginning of the systemic inflammatory response [35, 36]. Neutrophils accumulation in intracerebral hematoma can damage the brain parenchyma and disrupt the blood-brain barrier by releasing inflammatory factors, such as reactive oxygen species, matrix metalloproteinases, cathepsin and neutrophils extracellular trap (NETs) [37]. Additionally, neutrophil-mediated inflammation plays an important role in delayed cerebral vasospasm in aSAH; several studies have reported that NETs are involved in the occurrence and exacerbation of microthrombosis and microvasospasms [38, 39].

Monocytes are also important components of the innate immunity, involved in and regulating inflammatory responses, and play an important role in brain injury secondary to intracerebral hemorrhage [40]. After subarachnoid hemorrhage, the number of peripheral blood monocytes increased and entered the hematoma area in the form of macrophages to participate in local and systemic inflammatory response [41]. Monocyte-derived macrophages also respond to danger-associated molecular patterns by producing reactive oxygen species, cytokines, and chemokines, directly disrupting the blood-brain barrier integrity [42]. This vicious cycle of inflammation is currently believed to be involved in almost all pathophysiological mechanisms during aSAH, including delayed cerebral ischemia and cardiopulmonary impairment [43, 44].

Following aSAH, activation of the sympathetic pathways and the hypothalamic-pituitary-adrenal axis leads to the release of large amounts of catecholamines. These catecholamines promote the production and release of chemokines and cytokines, which in turn induce a systemic inflammatory response and post-stroke immunosuppression [45, 46]. The systemic inflammatory responses further stimulates an increase in neutrophils and monocytes in peripheral blood. Lymphocytopenia is a hallmark of neurogenic immunosuppression. The proliferation of inflammatory cytokines may promote the secretion of corticotropin-releasing hormone by the hypothalamic paraventricular nucleus after aSAH, leading to secretion of adrenocorticotropic hormone and glucocorticoids, which causes lymphocytopenia [46]. In patients with brain injury of various etiologies, a reduction in peripheral blood lymphocytes is associated with a significant deterioration of neurological outcomes and an increased risk of pulmonary infections [39, 43].

Excessive systemic inflammatory response and massive release of catecholamines not only contribute to secondary brain injury but also play an critical role in cardiopulmonary function impairment after aSAH [29, 47, 48]. Sympathetic overstimulation and massive release of peripheral circulatory inflammatory factors disrupt the integrity of pulmonary capillary endothelium and increase pulmonary vascular hydrostatic pressure, resulting in neurogenic pulmonary edema [35, 48]. Interstitial proteiniceous pulmonary edema is a hallmark of neurogenic pulmonary edema, which ultimately leads to pulmonary ventilation disorders [49]. In addition, sympathetic storms impair cardiomyocyte function, affect cardiopulmonary hemodynamics, and lead to changes in ventilate flow ratio. Increased pulmonary vascular permeability promotes the accumulation of neutrophils in the lung parenchyma, reducing lung compliance [50].

Local inflammation of intracranial primary brain injury is difficult to directly evaluate in clinical settings, and the changes of inflammatory cells in peripheral blood reflect systemic validation changes and intracranial local inflammation to some certain extent [49]. Due to the simple structure, a single indicator of inflammation is not enough to reflect the severity of inflammation [35]. SIRI, which was obtained by combining neutrophils, monocytes, and lymphocytes in a single marker, has been confirmed as a novel predictive inflammatory marker of DE in our cohort with a cutoff of 8.14. Elevated SIRI levels reflect proinflammatory hyperactivity (mediated by elevated neutrophils and monocytes), as well as anti-inflammatory suppression (caused by decreased lymphocytes) [51].

Another identified risk factor for DE after surgery for aSAH was lactic dehydrogenase in our study. Lactic dehydrogenase is a critical enzyme in the anaerobic metabolic pathway and is mainly distributed in the cytoplasm and mitochondria of various tissues, including brain tissue, and is released into the extracellular space after tissue damage, resulting in elevated serum levels [52]. Serum lactate dehydrogenase content is widely considered as a marker for evaluating tissue and cell damage. Serum lactate dehydrogenase levels have been reported as a unique and significant clinical biomarker for a variety of serious diseases, including severe inflammatory disease and stroke, in previous studies [53–55]. Additionally, elevated serum lactate dehydrogenase levels have been shown to be significantly associated with primary or secondary lung parenchymal damage [56]. Lactate dehydrogenase levels not only indicate the extent of tissue damage, but also be reported as a prognostic biomarker for inflammatory immune surveillance [57]. In our study, elevated serum lactate dehydrogenase was found to be one of the independent risk factors for DE after surgery of aSAH. Possible mechanisms include damage to brain cells or also the destruction of lung tissue cells by neurogenic pulmonary edema.

In the present study, We found that pre-operation SIRI predicted DE quite well in aSAH patients who underwent surgical operations. The peripheral blood cell test is the most commonly used test in clinical applications and can be accurately detected in primary care Settings. Thus, this novel, more comprehensive immuno-inflammatory marker has the potential to serve as a cost-effective and readily available risk stratification tool that can fully reflect the balance of inflammation versus immune response in patients. However, there are some limitations that need to be clarified. First, despite including data from 413 patients, this remains a single-center retrospective study with a relatively small size, which may introduce potential biases. Second, although we attempted to exclude factors that might influence the results, including potential inflammation, immunity, and respiratory comorbidities, these potential confounders may not be easily detected. Third, the study population was restricted to elderly patients aged ≥ 60 years, which limited the generalizability of the results to some extent. Fourth, the causes of postoperative DE are complex. We only analyzed the possible influencing factors before surgery and did not further analyze the related factors after surgery. Fifth, the modified Fisher grade is a semi-quantitative indicator of aSAH and may differ from the accurate aSAH volume. Finally, we only measured baseline SIRI at admission; the relationship between the dynamic changes of SIRI and the development of DE warrants further study.

## Conclusions

Preoperative SIRI is an independent risk factor for postoperative DE in elderly patients undergoing neurosurgical operation for aSAH. Evaluating the initial SIRI is useful for risk stratification of DE after surgery for aSAH.

## Data Availability

The data and code that support the findings of this study are available from the corresponding author upon reasonable request.

## Declarations

We confirmed that the manuscript complies with all instructions to authors. The authorship requirements have been met, and the final manuscript was approved by all authors. This manuscript has not been published elsewhere and is not under consideration by another journal.
